# Impact of Postmaneuver Sleep Position on Recurrence of Benign Paroxysmal Positional Vertigo

**DOI:** 10.1371/journal.pone.0083566

**Published:** 2013-12-18

**Authors:** Shufeng Li, Liang Tian, Zhao Han, Jing Wang

**Affiliations:** Department of Otolaryngology – Head and Neck Surgery, EYE & ENT Hospital of Fudan University, Shanghai, China; Imperial College London, United Kingdom

## Abstract

**Background:**

The necessity of postural restriction to patients suffering from benign paroxysmal positional vertigo is controversial.

**Objective:**

To investigate the impact of the sleep position after the repositioning maneuver on BPPV recurrence.

**Methods:**

150 unilateral BPPV patients who were treated by repositioning maneuver were distributed into two groups. The patients in group A were instructed to sleep in a semi-sitting position at an angle of approximately 30 degrees and refrain from sleeping on their BPPV affected side for one week. The patients in group B were told to sleep in any preferred position. The comparison of recurrence rates according to different actual sleep positions in one week and one month was performed.

**Results:**

There was a statistically significant correlation between the sleeping side and the side affected by BPPV. Without instructions on postural restriction, most patients (82.9%, 73/88) avoided sleeping on their affected side. The patients sleeping on their affected side had a higher recurrence rate (35.3%) than ones sleeping in other positions in the first week after the repositioning maneuver (*p*<0.05, Chi-square test and Fisher's exact test). The patients sleeping randomly in following 3 weeks had a lower recurrence rate than ones sleeping in other position (*p*<0.05, Fisher's exact test).

**Conclusions:**

BPPV patients had a poor compliance to postural instructions. The habitual sleep side was associated with the side affected by BPPV. The patients sleeping on their affected side had a higher recurrence rate than those sleeping in other positions in first week after the repositioning maneuver.

## Introduction

Benign paroxysmal positional vertigo (BPPV) is the most common cause of vertigo encountered in otology and neurology clinics. The characteristic clinical presentation of temporary vertigo attacks that lasts for seconds is generally believed to be caused by the dislocation of the otoconia in the semicircular canals [1], [2], detaching from the macula of the utricle [3]. Several maneuvers have been proposed to treat BPPV that yield high success rates [4]–[6]; however, there is a significant recurrence rate of 13.5 percent at 6-month follow-up [7], [8]. The recurrence rate at 1 year after treatment was 10 to 18 percent [9], [10]. Patients suffering from BPPV after trauma or with ipsilateral Meniere's disease have been shown to have an even higher BPPV recurrence rate [11], [12].

Postural restrictions after performing repositioning maneuvers were supposed to be able to improve the treatment outcome and prevent the recurrence of BPPV. Therefore, postural restriction was suggested in early description of the repositioning maneuver therapy for BPPV [4]. However, there is no agreement on the effectiveness or standard protocol of postural restriction. Most studies on the efficiency of postural restrictions did not show a difference in the recurrence rates between the groups with or without postural restrictions [13]–[17]. In addition, postural restriction might cause some discomfort and inconvenience to patients and affect their social life and sleep quality. On the contrary, Cakir et al. reported that the difference between restricted and non-restricted groups in the number of maneuvers required for treatment was statistically significant [18]. Some studies also showed a better outcome for patients with postural restrictions but did not detect a statistically significant difference due to small sample sizes [13], [19]–[21]. However, patients' compliance to postural restriction has not been seriously considered in most of these studies and may affect their results. In addition, more and more authors suggested the sample sizes in these studies were not sufficient to detect a significant difference and multicenter research with the adoption of improved methodology is still necessary to determine the contribution of postural restriction to the prevention of recurrence [13], [22], [23].

In this study, we investigated the compliance to postural restriction in BPPV patients and the relationships between the habitual sleep position and the side affected by BPPV as well as the impact of the sleep position after the repositioning maneuver on BPPV recurrence.

## Materials and Methods

From October 2013 to December 2013, a prospective study was conducted at a university-affiliated tertiary hospital. This study was approved by Ethical Board of EYE & ENT Hospital of Fudan University. Written consent was not concluded to be unnecessary because the data were analyzed anonymously and waived by Ethical Board. Oral consent were obtained from all patients included in this study. They were told they would be involved a clinical study on the relationship of BPPV recurrence and postmaneuver sleep position. The diagnosis criteria followed the guideline provided by the American Academy of Otolaryngology - Head and Neck Surgery [24]. All the patients included in this study had a history of rotatory vertigo that lasted for seconds. They all had rotatory vertigo with direction-changing reversible torsional or geotropic horizontal nystagmus triggered by Dix-Hallpike tests or supine roll tests, respectively. These diagnostic procedures were performed on a TRV chair (CTRV INNOVATION, Ventabren, France), which was a rotary chair that could make the patients rotate on the plane of the semicircular canals [25]. The repositioning maneuvers were then performed if the results of the test were positive. For posterior semicircular canal BPPV (P-BPPV), the patients were tilted backward and rotated 360 degrees at the axis of the corresponding canal. The rotations started from the position of 120 degrees and continued in 60-degree steps until the sitting position was reached. For the geotropic type of lateral semicircular canal BPPV (L-BPPV), the side on which the patients had stronger attack of vertigo and nystagmus was concluded as the affected side. The patients were treated by rotating from the supine position towards healthy side and continued in 90-degree steps until reaching the affected side, which was similar to the Lempert maneuver [26]. For the apogeotropic type of L-BPPV, the rotations were performed in a contrary direction to geotropic type of L-BPPV.

All patients included in this study showed complete relief of vertigo and nystagmus immediately after the repositioning maneuver and had no vertigo attack in 48 hours. They were randomly distributed into two groups. The patients in group A were instructed to sleep in a semi-sitting position at an angle of approximately 30 degrees and refrain from sleeping on their affected side for one week. They were also told to sleep in their preferred positions after one week. The patients in group B were told to sleep in any preferred position.

These patients were followed up at one week and one month after the repositioning maneuver. They were asked about their actual sleep positions during the first week and during the 2–4 weeks after the repositioning maneuver as well as the recurrence of rotatory vertigo lasting for seconds. Finally, the patients were distributed into five groups according to their actual sleep positions in first week after the repositioning maneuver: the affected side, healthy side, random, supine and semi-sitting position.

The Chi-square test and Fisher's exact test, if expected frequencies were less than 5, were used for statistical analyses, and *P*<0.05 was considered significant.

## Results

A total of 150 patients, 48 men and 102 women, were diagnosed with unilateral BPPV and treated with the repositioning maneuver. The male-female incidence ratio was 0.471∶1. The patients' ages ranged from between 23 to 80 years old, with an average age of 53.2 years old. The group with the most subjects was women in the fifth decade of life. The patients' diagnoses included 102 cases of unilateral P-BPPV (68.0%), 31 cases of unilateral L-BPPV (20.7%), 2 cases of unilateral superior canal BPPV (1.3%) and 15 cases of unilateral multiple-canal BPPV (10.0%).

There were 72 cases in the group A of restriction of sleep position and 78 cases in the group B of no restriction of sleep position. The relapse rates in one week were 9.7% (7/72) in group A and 16.7% (13/78) in group B, respectively. There was no significant difference between the two groups (*P*>0.05, Chi-square test). The relapse rates in first month after the repositioning maneuver were 19.4% (11/72) in group A and 24.4% (14/78) in group B, respectively, which was also not significantly different (*P*>0.05, Chi-square test). (Table 1)

**Table 1:** The comparison of recurrence rates in patients who were with (group A) or without (group B) the instruction of postural restriction.

Group	Number of patients with recurrence in the first week (rate)	Number of patients with recurrence in the first month (rate)	Number of treated patients
A	7 (9.7%)	14 (19.4%)	72
B	13 (16.7%)	19 (24.4%)	78
Total	20(13.3%)	33(22.0%)	150

*P*>0.05, Chi-square test). There was no significant difference between the two groups (

However, most patients (82.1%, 64/78) who were not instructed to sleep in a restricted position did not sleep in a random position but, rather, had an obvious preference to avoid sleeping on their affected side during the first week after the repositioning maneuver. In other words, most patients without the instruction of postural restriction actually imposed a sleep-position restriction on themselves. At the same time, some patients (41.7%, 30/72) with the instruction of postural restriction did not completely take the semi-sitting position during the first week after the repositioning maneuver. Thus, we analyzed the relationships between the recurrence rate of BPPV and the actual sleep positions instead of the instructed sleep position.

The patients with unilateral BPPV in this study were divided into 5 groups by their sleep position during the first week after the repositioning maneuver, including affected side, healthy side, supine, random and semi-sitting position at approximately 30 degrees (Table 2). No difference among groups was found to be correlated with age or sex. There was a higher recurrence rate of 31.3% in patients sleeping on their affected side than in patients sleeping in other positions in one week after repositioning maneuver (*P*<0.05, Chi-square test and Fisher's exact test). In addition, there was no significant difference between the recurrence rate in a semi-sitting position at 30 degrees and rates in other positions, except for the affected side (*P*>0.05, Chi-square test and Fisher's exact test). The patients without recurrence in the first week were divided into three groups, including affected side, random position and unaffected position (Table 3). The patients sleeping on their affected side did not have a higher recurrence rate than patients sleeping in other positions during the 2–4 weeks after the repositioning maneuver (*P*>0.05, Fisher's exact test). However, patients who took a random sleep position had a lower recurrence rate than patients sleeping in other positions (*P*<0.05, Fisher's exact test).

**Table 2:** The comparison of recurrence rates in patients who slept in different positions during the first week after the repositioning maneuver.

Sleep position in one week	Number of patients with recurrence (rate)	Number of patients without recurrence (rate)	Number of treated patients
Affected side	5 (31.3%)	11	16
Random	4 (13.3%)	26	30
Supine	3 (10.3%)	26	29
Unaffected side	4 (12.1%)	29	33
Semi-sitting at 30 degrees	4 (9.5%)	38	42
Total	20 (13.3%)	130	150

% in patients sleeping on their affected side than in patients sleeping in other positions in one week after repositioning maneuver (*P*<0.05, Chi-square test and Fisher's exact test). In addition, there was no significant difference between the recurrence rate in a semi-sitting position at 30 degrees and rates in patients sleeping in other positions, except for the affected side (*P*>0.05, Chi-square test and Fisher's exact test). There was a higher recurrence rate of 31.3

**Table 3:** The comparison of recurrence rates in patients who slept in different positions during the 2–4 weeks after the repositioning maneuver (excluding the recurrent patients in one week after repositioning maneuver).

Sleep position	Number of patients with recurrence (rate)	Number of patients without recurrence (rate)	Number of treated patients
Affected side	10 (15.2%)	56 (84.8%)	66
Random position	0 (0)	48 (100%)	48
Unaffected side	3 (18.8%)	13 (81.2%)	16
Total	13 (10%)	117 (90.0%)	130

–4 weeks after the repositioning maneuver (*P*>0.05, Fisher's exact test). The patients who took a random sleep position had a lower recurrence rate than patients sleeping in other positions (*P*<0.05, Fisher's exact test). The patients sleeping on their affected side did not have a higher recurrence rate than patients sleeping in other positions during the 2

Of 150 patients diagnosed with unilateral BPPV, 86 (57.3%) patients had a habit of sleeping laterally, 31(36.0%) on their left side and 55 (64.0%) on their right side (Table 4). Of the 31 patients who slept on their left side, 20 patients (64.5%) were diagnosed with left BPPV. Meanwhile, of the 55 patients who slept on their right side, 39 patients (70.9%) were diagnosed with right BPPV. There was a statistically significant correlation between the habitual sleeping side and the side affected by BPPV (*P*<0.05, Chi-square test).

**Table 4:** The relationship between the side affected by BPPV and the habitual sleep position.

Sleep position	Diagnosis										Total (%)
	Left side					Right side					
	P	L	PL	S	Total (%)	P	L	PL	S	Total (%)	
Left side	11	8	0	1	20(64.5)	8	2	1	0	11(35.5)	31(100)
Right side	15	1	0	0	16(29.1)	27	4	8	0	39(70.9)	55(100)
Supine	5	4	0	0	9(45.0)	9	2	0	0	11(55.0)	20(100)
Random	9	7	1	1	18(40.9)	20	4	2	0	26(59.1)	44(100)
Total	40	20	1	2	63(42.0)	64	12	11	0	87(58.0)	150(100)

P, posterior canal BPPV; L, lateral canal BPPV; PL, posterior canal BPPV and lateral canal BPPV; S, superior canal BPPV.

*P*<0.05, Chi-square test). There was a statistically significant correlation between the habitual sleeping side and the side affected by BPPV (

## Discussion

Our study showed that there was a statistically significant correlation between the preferred sleep side and the affected side of BPPV. This result is consistent with other reports [27]–[31]. It was also suggested that postoperative bed-rest may facilitate the development of BPPV[32]. Based on this finding, it is reasonable to assume that continuous lateral positioning of the head facilitates the detachment of the otoconia or its migration into the semicircular canals of the undermost ear due to gravity. Therefore, proper postural restriction after the repositioning maneuver should be helpful to decrease the chance of free-floating otoconial particles returning into the canals, and, thus, increase the treatment outcome and prevent recurrence of BPPV.

Postural restriction was suggested at the beginning of the repositioning maneuver therapy for BPPV [4]. However, the necessity of postural restriction after the repositioning maneuver is controversial. Most relevant studies did not support the effect of postural restriction on the treatment outcome and recurrence rate of BPPV. On the contrary, Cakir et al. reported that the difference between restricted and non-restricted groups in the number of maneuvers required for treatment was statistically significant [18]. Some studies also showed a better outcome for patients with postural restrictions but did not detect a statistically significant difference due to small sample sizes [13], [19]–[21]. A recent meta-analysis included 9 relevant articles and concluded that there were no significant differences between patients instructed to restrict their posture after the repositioning maneuver and patients allowed to move freely after a repositioning maneuver with regard to the presence or absence of post-maneuver symptoms[23]. However, the sample sizes in these studies were not sufficient to detect a significant difference. It was suggested that more patients (200) should be recruited to gain statistical significance because the detected clinical difference (15.8%) is small compared to the difference set during the designing of the protocol (30%) [13]. Fortunately, more and more authors have become aware of these issues and have indicated that multicenter research with the adoption of improved methodology is still necessary to determine the contribution of postural restriction to the prevention of recurrence [13], [22], [23].

We proposed that the effectiveness of postural restriction is limited by the poor compliance of patients. As suggested by our study, the compliance to postural restriction was quite poor. Most patients (82.1%) in the group of non-restricted sleep position avoided sleeping on their affected side. This is consistent with a previous report that found that patients with BPPV tend to avoid provoking head positions in fear of experiencing vertigo [33]. Although it is reported that people have a tendency to maintain their initial sleeping position until they wake up [34], some patients in the group of restricted sleep position actually did not follow the instruction because of the discomfort and inconvenience of the instructed position. In addition, older patients might change to their position to lie on their right side because of cardiovascular conditions [35]. However, patient compliance and satisfaction to postural restriction were not evaluated or indicated in almost any of the studies [13]–[17], [23]. Interestingly, in a study including a large number patients (n = 119) and excluding the patients in the control group who refrained from turning to the affected side during sleep or performing similar postural restriction, a statistically significant difference of treatment outcome between groups with or without postural restriction was found [18]. Moreover, in two studies that reported that postural restriction did not affect the success of the repositioning maneuver, the authors recommended avoidance of sudden head movements, thus causing unplanned crossover between the two groups [15], [36].

Our study demonstrated that sleep position after a repositioning maneuver has statistically significant impact on the recurrence rate of BPPV. The patients sleeping on their affected side after the repositioning maneuver had a higher recurrence rate than patients with other sleep positions. However, other studies did not find an effect of postural restrictions on recurrence of BPPV [14]–[16], [18], [22]. Another study showed that the time course in remission of positional vertigo is not affected by the head-lying side during sleep [31]. The difference between the results of our study and others might be partly due to the difference in follow-up duration along with sleep position. Unlike other studies that evaluated the recurrence rate for a long duration after postural restriction, this study evaluated the recurrence rate along with different sleep positions at one week as well as one month after the repositioning maneuver. Although short-term postural restriction could reduce the long-term recurrence rate of BPPV, refraining from sleeping on the affected side after the repositioning maneuver might reduce the recurrence rate during the period with sleep-position restriction.

To our knowledge, it is still unknown how otoconia detaches from the utricle and what happens to the otoconia after they are replaced into the utricle. They might dissolve in endolymph or be reabsorbed into the macule [23]. It was reported that otoconial debris of the frog was able to dissolve in normal endolymph very rapidly (in approximately 20 hours) [37]. In another study, frog otoconial mass was found to become stabilized several minutes after being placed on the utricular macular otoconia [38], and, therefore, the replaced otoconia should no longer been able to play a role in BPPV recurrence. BPPV recurrence might be caused by the newly detached otoconia but not the replaced otoconia. Continuously sleeping on the affected side would increase the chance of newly detached otoconia moving into the undermost canals due to the effect of gravity, which then causes BPPV recurrence. Changing the sleep position randomly would facilitate the random moving of the otoconia and decrease the chance of the otoconia getting into the undermost canals. These speculations were proved by our study.

The protocols of postural restriction are varied in the literatures, but usually include keeping the head upright for 48 h and refraining from lying on the affected side for at least one week [4], [39]–[41]. Our study indicated that sleeping in a semi-sitting position at 30 degrees did not decrease the recurrence rate. Sleeping on the affected side increased the recurrence rate of BPPV at one week after repositioning maneuver. The patients changing the position randomly during sleep had a much lower recurrence rate than patients in other positions. Therefore, BPPV patients after the repositioning maneuver should be advised not to sleep on their affected side and be encouraged to sleep in a random position.

It should be noted that the data of postmaneuver sleep position were reported by the patients and their partners or families in our study and in previous studies on the impact of postural restriction on BPPV recurrence [13]–[15], [17]–[19], [22], [23], [27], [31]. The employment of proper monitoring device in future study might be helpful to acquire more accurate data of postmaneuver sleep position and to maintain the restricted position after repositioning maneuver.

Taken together, BPPV patients had a poor compliance to the postural instructions. The habitual sleep side of patients suffering BPPV was associated with their affected side of BPPV. The patients sleeping on their affected side had a higher recurrence rate than those sleeping in other positions at one week after the repositioning maneuver. BPPV patients should be advised to refrain from sleeping on their affected side for at least one week after the repositioning maneuver.
